# Chromosome-Wide Characterization of Intragenic Crossover in Shiitake Mushroom, *Lentinula edodes*

**DOI:** 10.3390/jof7121076

**Published:** 2021-12-15

**Authors:** Wenbing Gong, Nan Shen, Lin Zhang, Yinbing Bian, Yang Xiao

**Affiliations:** 1 Institute of Applied Mycology, Huazhong Agricultural University, Wuhan 430070, China; gongwenbing@caas.cn (W.G.); bioshennan@foxmail.com (N.S.); cai284829597@126.com (L.Z.); bianyinbing@mail.hzau.edu.cn (Y.B.); 2 Institute of Bast Fiber Crops, Chinese Academy of Agricultural Sciences, Changsha 410205, China

**Keywords:** *Lentinula edodes*, intragenic crossover, hotspots, *cis*- and *trans*-loci

## Abstract

Meiotic crossover plays a critical role in generating genetic variations and is a central component of breeding. However, our understanding of crossover in mushroom-forming fungi is limited. Here, in *Lentinula edodes*, we characterized the chromosome-wide intragenic crossovers, by utilizing the single-nucleotide polymorphisms (SNPs) datasets of an F_1_ haploid progeny. A total of 884 intragenic crossovers were identified in 110 single-spore isolates, the majority of which were closer to transcript start sites. About 71.5% of the intragenic crossovers were clustered into 65 crossover hotspots. A 10 bp motif (GCTCTCGAAA) was significantly enriched in the hotspot regions. Crossover frequencies around mating-type A (MAT-A) loci were enhanced and formed a hotspot in *L. edodes*. Genome-wide quantitative trait loci (QTLs) mapping identified sixteen crossover-QTLs, contributing 8.5–29.1% of variations. Most of the detected crossover-QTLs were co-located with crossover hotspots. Both *cis*- and *trans*-QTLs contributed to the nonuniformity of crossover along chromosomes. On chr2, we identified a QTL hotspot that regulated local, global crossover variation and crossover hotspot in *L. edodes*. These findings and observations provide a comprehensive view of the crossover landscape in *L. edodes*, and advance our understandings of conservation and diversity of meiotic recombination in mushroom-forming fungi.

## 1. Introduction

Meiotic recombination is an essential cellular process shared across eukaryotes, which recombines genetic variation and plays a pivotal role in genome evolution [1,2]. During meiosis, DNA double-strand breaks (DSBs) enter interhomolog repair to yield two types of recombination products, crossovers and non-crossovers [2,3]. Meiotic crossovers between homologous chromosomes break-up haplotypes and shuffle genetic variations, and are essential for variety breeding, which relies on the ability to combine favorable alleles [4]. Thereby, the profound understanding of molecular mechanisms driving meiotic crossovers has the potential to accelerate variety breeding and allow the more effective use of the available variation [5].

The occurrence of meiotic crossovers along chromosomes is highly non-random. Crossovers are preferentially located in kilobase-sized regions known as crossover hotspots [6]. During the past decades, the meiotic recombination process has been extensively studied in model species and has provided important insights into conserved mechanisms [6,7]. Meanwhile, meiotic crossovers vary considerably between species, populations and individuals, and the regulations of crossover variation are under very complicated *cis*- and *trans*-genetic control [8,9,10]. Unique and novel meiotic crossover features have also been observed in mushroom-forming basidiomycetes. For instance, in a hypervariable basidiomycete *Schizophyllum commune*, meiotic crossover preferentially occurred in regions of high local similarity [11]. Extremely, in a homothallic basidiomycete *Agaricus bisporus*, meiotic crossovers were almost restricted exclusively to regions of about 100 kb at the chromosome ends [12]. The extensive investigation of meiotic crossover in mushroom-forming basidiomycete may help to shape our understanding of conservation and diversity in meiotic strategies.

*Lentinula edodes*, also called the shiitake mushroom, is renowned for its compelling health-promoting properties [13]. Currently, *L. edodes* is widely cultivated and it contributes to about 22% of the world’s total mushroom production [14]. Given its importance in research and the food industry, *L. edodes* has been extensively studied as a model basidiomycete for mushroom genetics and physiology research. Recently, we re-assembled a chromosome-level genome and genotyped an F_1_ haploid progeny by high-throughput RNA-seq [15]. These F_1_ haploid progenies were derived from products of one generation of meiosis and were therefore suitable to dissect meiotic crossover events. Herein, we re-mapped the single-nucleotide polymorphisms (SNPs) into the linkage map of *L. edodes*, and identified genome-wide intragenic crossovers by haplotype switches along the chromosomes. The crossover hotspots and genetic architecture of crossover variation in these F_1_ haploid progenies were also characterized. Findings in this study may advance our understandings of meiotic recombination in mushroom-forming basidiomycetes.

## 2. Materials and Methods

### 2.1. Haploid Progeny Population

The F_1_ haploid progenies, comprising 110 single-spore isolates (SSIs) of the hybrid strain L205-6 × W1-26, were generated and utilized previously [15,16]. Genomes of the two parent monokaryons L205-6 and W1-26 had been re-sequenced [17]. Recently, via high-throughput RNA-seq and genotypes deduction, we genotyped the 110 SSIs and constructed a high-density genetic map, Lemap2.0, comprising 488 recombination bins [15]. A 37.23 Mb genome sequence of *L. edodes* was also re-anchored into nine pseudo-chromosomes (https://mycocosm.jgi.doe.gov/Lenedo1, accessed on: 12 May 2021), which aligned into nine linkage groups of Lemap2.0 [15]. All the sequencing data are deposited into GenBank under the accession of PRJNA649389 (https://www.ncbi.nlm.nih.gov/bioproject/PRJNA649389, accessed on: 22 November 2021).

### 2.2. Identification of Crossovers and Hotspots

In order to identify crossovers, we re-mapped all 69,634 markers (SNPs and InDels) into Lemap2.0 [15]. According to the recombination studies in plants [9,18], crossover events (recombination breakpoints) were identified by the changes in the parental genotype phase ordered along the chromosomes. A python script “findCrossover.py” (Supplementary File S1) was written to count the number of crossover events per chromosome for each individual. To reduce the influence of mis-genotyped and misplaced markers on identification of crossovers, one crossover event was recorded in a case where at least five consecutive markers on both sides of the recombination breakpoint were from different parents [19]. Meiotic crossovers do not occur uniformly along the chromosomes. In this study, we divided the *L. edodes* genome into non-overlapping 10 kb windows and recorded crossovers in each window. To identify crossover hotspots, the Poisson function in Excel was used to find the threshold value of the number of crossovers in each 10 kb window [20,21]. Then, the motif enrichment analysis in crossover hotspots was performed using the Homer findMotifsGenome module in Homer software v4.10.1 [22].

### 2.3. Quantitative Trait Loci (QTLs) Mapping of Crossover Variation

In order to detect genetic factors conferring crossover variation in *L. edodes*, total number of crossover events (TCO), crossover events in hotspots (HCO) and crossover events of each chromosome (COchr) were used as the phenotype dataset for QTL analysis. Combining Lemap2.0, genotypic data and phenotypic data of the 110 SSIs, QTLs underlying crossover variation were mapped using composite interval mapping with WinQTLCart 2.5 [23]. Parameters for QTL detection were set to model 6 with a scanning step of 1 cM (number of control markers = 5; window size = 10 cM). The 1000-permutation-based LOD threshold was calculated to declare the presence of a QTL (*p* < 0.05). Confidence intervals (CIs) of QTLs were supported by LOD peak minus one. Detailed descriptions (position, additive effect, LOD score, CI and percentage of phenotypic variation explained) were provided to depict each QTL [17]. For the QTL mapping of crossover variation on each chromosome, if QTL was located in the chromosome, of which the crossover number was analyzed, such QTL was defined as *cis*-prone crossover QTL. If QTL is not mapped on the chromosome, of which the crossover number was analyzed, the QTL was defined as *trans*-crossover QTL. Genes in the CIs of QTLs were analyzed to screen for candidate genes regulating crossover variation.

## 3. Results and Discussion

### 3.1. Distribution of Crossovers in Haploid Progenies

In previous work, crossover events were extensively quantified by re-sequencing the recombined gametes derived from meiosis, such as in human [24], yeast [25], Arabidopsis [26] and also in fungi [11]. Here, the 110 F_1_ SSIs of *L. edodes* were the recombined basidiospores derived from meiosis. Therefore, the crossover events could be scored by counting the recombination breakpoints, in which change of linkage phase between markers indicates the occurrence of a crossover [5]. Moreover, to avoid false crossover detection, a rigorous criterion for haplotype switches was employed. We define a crossover as a change in parental genotype phase for at least five consecutive markers [19]. 

The feature of crossover distribution in *L. edodes* was summarized in Supplementary Table S1. In the 110 F_1_ meiotic SSIs, a total of 884 intragenic crossovers (TCOs) were identified. Results of the number of crossovers suggested that limited crossovers occurred per chromosome per individual, varying from 0.25 to 1.80 (Supplementary Table S1). The low frequency of crossovers per chromosome might be due to crossover interference, i.e., crossover in one genetic interval reducing the likelihood of a co-occurring crossover in an adjacent interval [6,27]. Here, in *L. edodes*, there was a sizeable proportion of progenies that did not show crossover, which was in line with *Zymoseptoria tritici* [28]. The occurrence of achiasmatic chromosomes was also observed in mushroom-forming basidiomycetes, such as *Hericium erinaceus* [19] and *Pleurotus ostreatus* [29]. Overall, the low number of crossovers may limit the efficiency of the breeding process [4]. 

The number of crossovers per SSI ranged from 4 to 16, showing continuous distribution, and suggesting their nature of quantitative traits (Supplementary Figure S1). Comparatively, the number of crossovers per individual in *L. edodes* (average 8.04) was on the same order of magnitude as other heterothallic basidiomycetes, such as 10.4 in *H. erinaceus* [19] and 4.2 in *S. commune* [11], but larger than the homothallic basidiomycete *A. bisporus*, in which only an average of 0.5 crossovers per individual was observed [12]. Similar to *H. erinaceus* [19], the number of crossovers was related to the chromosome length, and long chromosomes tended to have a higher number of average crossovers (Supplementary Table S2).

Among these 884 intragenic crossovers, the majority of intragenic crossovers (59.7%) were closer to transcript start sites, which was consistent with the pattern observed in yeast [30] and maize [8], but differs from recombination studies in humans, where crossovers decreased towards gene transcripts [31]. This may indicate that in *L. edodes*, recombination-driven genomic exchanges tend to alter the promoter or regulatory regions of genes.

### 3.2. Crossover Hotspots and Mating Type

**Figure 1:**
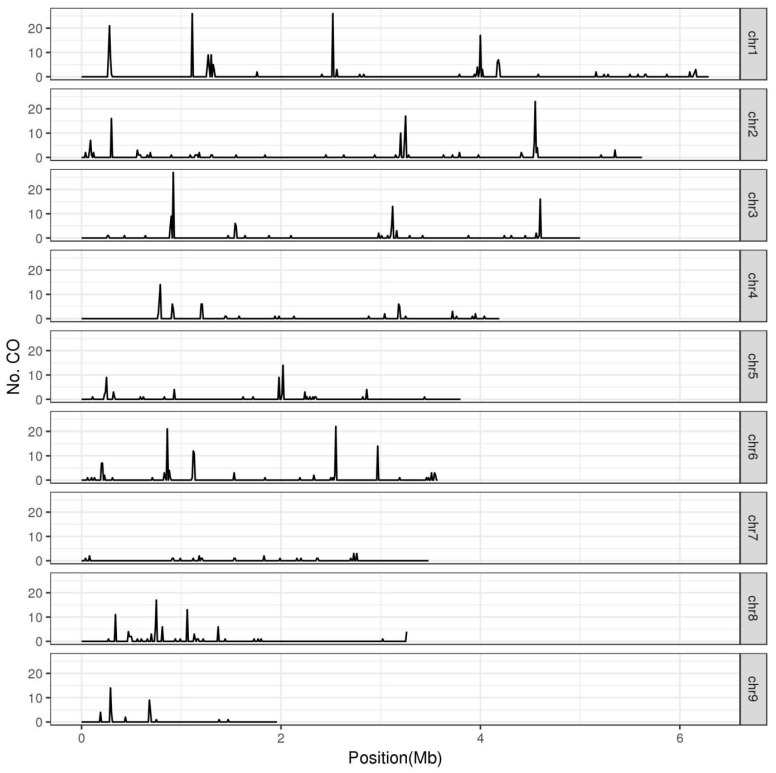
Distribution of crossover events along the chromosomes of *L. edodes*. The *L. edodes* genome was divided into non-overlapping 10 kb windows and recorded the crossover events of each window. The regions, for which no less than four crossovers per 10 kb segment were identified as crossover hotspots.

In most eukaryotes, crossovers are non-uniformly distributed along chromosomes and typically cluster within hotspots [32]. It has been widely reported in fungi that crossovers preferentially located near telomeres and sub-telomeric regions [12,19,28,29]. Here, in *L. edodes*, we recorded crossovers in non-overlapping 10 kb segments and identified hotspot regions with large numbers of crossovers (Figure 1). In these F_1_ haploid progenies, an average of 0.23 crossovers were recorded per 10 kb segments of the *L. edodes* genome. The regions with at least four crossovers per 10 kb segment were identified as crossover hotspots (Poisson distribution test, *p* < 0.0001). Thereby, we found 65 crossover hotspots in the *L. edodes* genome, where the crossovers reached to 17–117 times the genome average (Supplementary Table S3). These hotspots were scattered onto eight chromosomes, but not on chr7. Although the underlying genetic mechanism was unknown, the whole chr7 seemed to be recombination-suppressed (Figure 1). Recombination suppression of whole chromosomes was also clearly shown in *A. bisporus* [12,33]. The longest chromosome, chr1, had the most crossover hotspots (15 of 65; Supplementary Table S3). However, the largest number of crossovers was observed on the 10 kb hotspot region on chr3 (920–930 kb), which was 117-fold higher than the genomic background (Supplementary Table S3). These 65 crossover hotspots represented 1.7% of the genome, and contained 71.5% (632 of 884) of the intragenic crossovers. These 632 crossovers in hotspots were therefore considered as HCOs. HCO was significantly positively correlated with TCO (r = 0.745, *p* < 1.2 × 10^−20^). SNP frequency in crossover hotspots was higher than that of genome average (ANOVA, *p* < 0.006). In crossover hotspots, enrichment analysis also revealed a significantly enriched motif (GCTCTCGAAA) similar to the binding motif of SWI4, a transcription factor that is an essential part of a complex involved in cell-cycle-dependent transcription in yeast [34] (*p* < 1 × 10^−8^). The existence of crossover hotspots was in agreement with observations in other fungi: 1.4% of most active sites correspond to 32.3% of crossover in *H. erinaceus* [19] and 1.9–3.3% of the genome contained 27.8–38.2% of crossovers in *Z. tritici* [28]. Additionally, in yeast, 10% of the most active sites corresponded to more than 50% of all recombination events [30]. The distribution of crossovers in *L. edodes* seems to be more concentrated, which might be because they were intragenic crossovers.

Many sexually reproducing species distribute crossovers in a sex-specific pattern along chromosomes [35,36,37]. In fungi, recombination also plays critical roles in mating-type determination [38]. Here, in *L. edodes*, we tested the effect of mating type of SSIs on TCO and HCO. Weak differences of TCO and HCO were observed among the SSIs with different mating-type A (MAT-A) loci. The average TCO and HCO of the SSIs with MAT-A_1_ (same as the parent monokaryon L205-6) were slightly higher than those of MAT-A_2_ SSIs (same as W1-26) (Supplementary Figure S2). This result differed from that of *H. erinaceus*, in which no significant difference was observed among total crossovers of different mating-type SSIs [19]. Differences in viability, germination, or vegetative growth rate associated with different mating haplotypes may lead to selection bias of monokaryons [39], and further lead to crossover bias. Here, in the 110 SSIs of *L. edodes*, no distorted segregation was observed for MAT-A loci (MAT-A_1_-SSIs : MAT-A_2_-SSIs was 53 : 57). There seems to be other reasons for crossover bias between mating types. In animals and plants, extensive recombination differences between sexes are observed [36,37]. In basidiomycetes, whether and why there is crossover bias between mating types needs extensive research. Interestingly, the region surrounding the MAT-A loci formed a crossover hotspot (Hotspot20) in *L. edodes* (Supplementary Table S3), while that region was characterized by suppressed recombination in *H. erinaceus* [19]. In fact, although the evolutionary drivers are still unclear, contrasting relationships between recombination and mating-type determination are observed [38,40]. Regions involved in mating compatibility are in some cases associated with recombination suppression, while in other cases with enhanced recombination [40]. 

### 3.3. Regulation of Meiotic Crossovers

The regulation of meiotic crossovers is very complex and multilevel, including genome-wide mechanisms, chromosome-specific phenomena and local effects [6]. In recent years, great efforts have been made to increase the insight into recombination in model species, and several loci and genes that shape crossover variation have been identified [9]. Additionally, the QTL mapping method was employed to dissect the genetic mechanisms underlying crossover variation in animals and plants [9,18,41,42]. Here, in *L. edodes*, to decipher the genetic factors conferring the non-uniformity of crossovers, TCO, HCO and COchr were taken as phenotypic traits to perform QTL mapping. TCO indicated global crossover events across the genome, HCO evaluated crossovers in specific hotspot regions, while COchr would be more accurate to quantify crossovers for each chromosome.

**Figure 2:**
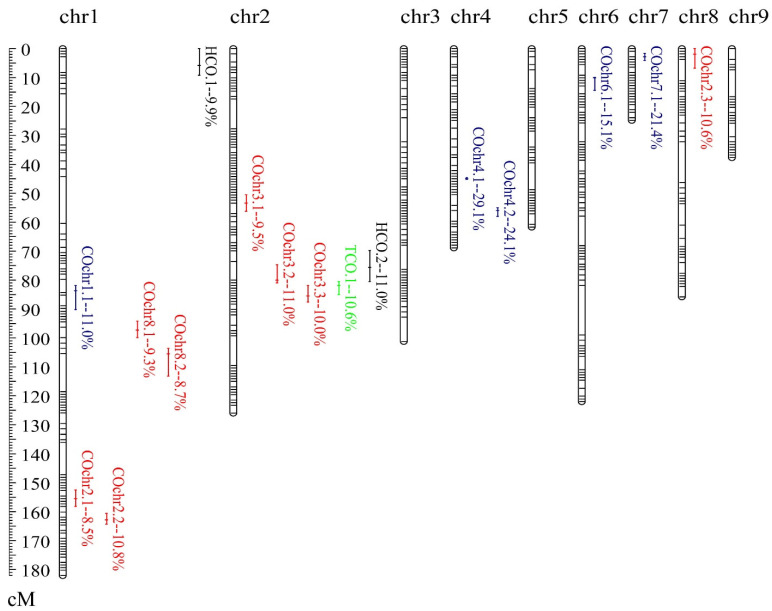
Distribution of QTLs for crossover variation in *L. edodes*. This Lemap2.0 was generated previously [15]. Sixteen QTLs for crossovers were shown on the right side of the chromosomes. The LOD-1 confidence interval was indicated by the length of the QTL bar, and the position of the LOD peak was represented by the short line in the middle of the QTL bar. The percentage value is represented the R^2^ value (percentage of explained phenotypic variation). The *cis-prone* QTLs are marked in blue, while *trans*-QTLs are in red. QTLs for HCO and TCO are marked in black and green, respectively.

In total, sixteen loci, including one TCO-QTL, two HCO-QTLs and thirteen COchr-QTLs, were mapped for crossover variation (Table 1, Figure 2). Similar to *H. erinaceus* [19], most of the detected crossover-QTLs were within or next to the crossover hotspots, suggesting conservation of the mechanism in basidiomycetes, i.e., genes in hotspots shaped crossover variation in hotspots, and further regulated the local and global crossover. The locus *tco.1* was mapped on chr2, contributing 10.6% of global crossover variation across the genome. For crossover events in hotspots, *hco.1* and *hco.2* accounted for 20.9% of HCO variation. The *tco.1* and *hco.2*, both showing negative additive effects, were close together on chr2, which suggests the existence of linked genes regulating TCO and HCO. The linkage between QTLs for TCO and HCO contributed to their phenotypic correlation.

For crossover variation of each chromosome, thirteen COchr-QTLs were distributed on six chromosomes, and both *cis* and *trans* factors contributed to local crossover variation. Of the thirteen COchr-QTLs, five were *cis-prone* QTLs, and eight were *trans*-QTLs (Table 1). An average of 20.1% of crossover number variation could be explained by *cis-prone* QTLs, which was higher than an average of 9.8% of *trans*-QTLs (*p* = 0.002). Of the *cis-prone* QTLs, the two adjacent loci *cochr4.1* and *cochr4.2* could explain up to 53.2% of the crossover variation of chr4. Additionally, the two QTLs were identified with high statistical power. The QTL *cochr7.1* also contributed to more than 20% of the phenotypic variation. With CI of 2.5 cM, the physical length of *cochr7.1* (836.7 Kb) covered nearly 24% of chr7. It was mainly due to the low recombination rate (7.1 cM/Mb) on chr7 [15]. For mapped *trans*-QTLs, most were distributed on chr1 and chr2. On chr1, four *trans*-QTLs were identified underlying COchr2 and COchr8. On chr2, three *trans*-QTLs for crossover variation of chr3 were clustered. Moreover, *cochr3.2* was co-located with *hco.2*, while *cochr3.3* was co-located with *tco.1*. These results indicated the presence of a QTL hotspot on chr2 (ranged from 69.8 to 87.6 cM), that regulates local and global crossover variations. Interestingly, this QTL hotspot underlying crossover variations was overlapped with the meta-QTL *pq2.1* responsible for fruiting body-related traits [15]. In plants, such as maize, the significant associations between recombination events and variation in gene expression and agronomic traits were also identified [43]. Our previous studies have identified 246 phenotype-specific expressed genes underlying fruiting body-related traits [15]. Here, we analyzed the correlation of TCO and HCO with the expression of the 246 genes, and identified 47 significant crossover association signals in 37 genes (Supplementary Table S4). We also analyzed the correlation between TCO and HCO with the surveyed fruiting body-related traits of the F_1_ haploid progenies. No significant correlation was observed among crossovers and fruiting body-related traits (data not shown). This might be due to the fact that only a small part of variation both for crossovers and fruiting body-related traits could be explained by this QTL hotspot.

The confidence intervals for the sixteen mapped crossover-QTLs ranged from 2.0 to 836.5 kb, and contained a few to hundreds of genes (Table 1, Supplementary Table S5). Some well-known recombination-related genes were also found in the CIs of the QTLs. For instance, the *Rad51* gene family is evolutionarily conserved in eukaryotes, and plays a critical role in double-strand break repair, replication stress and meiosis [44]. The homologous gene of *Rad51C* was identified in *cochr4.1* (Supplementary Table S5). Moreover, the gene for Rad51-associated protein Brh2 was found in the mapped QTL hotspot underlying crossover variations (*cochr3.3* and *tco.1*) (Supplementary Table S5). Brh2 works hand-in-hand with Rad51 to promote the repair of DNA by homologous recombination [45]. The *HD1* gene encoding homeodomain protein was also included in this QTL hotspot (*cochr3.2* and *hco.2*). Thereby, the co-localizations of mating-type genes, crossover-QTLs and crossover hotspots, may contribute to the mechanism whereby crossover frequencies around MAT-A loci were enhanced in *L. edodes.*

## 4. Conclusions

In summary, we elucidated the dynamics of intragenic crossover in F_1_ haploid progenies of *L. edodes*. The 65 crossover hotspots and genetic architecture of crossover variation were characterized here. Enhanced crossovers adjacent to mating-type loci in *L. edodes* were also observed. QTL mapping revealed both *cis*- and *trans*-loci contributing to the nonuniformity of crossover along chromosomes. A QTL hotspot on chr2 regulating local and global crossover variation and crossover hotspot was identified. The findings of this study advanced our understandings for the conservation and diversity of meiotic recombination in mushroom-forming fungi.

## Figures and Tables

**Table 1:** QTLs for crossover variation of *L. edodes.*

Locus	Trait	Chr	Type	Position (cM)	LOD	Additive	R^2^ (%)	^a^ CI (cM)	^b^ No. of Genes	^c^ Association with Hotspots	Length of CI (kb)
*cochr1.1*	COchr1	1	*cis-prone*	83.7	3.07	0.61	11.0	81.9–90.2	6	hotspot8	29.4
*cochr2.1*	COchr2	1	*trans*	155.5	2.95	−0.33	8.5	152.5–158.2	5	hotspot13	9.5
*cochr2.2*	COchr2	1	*trans*	162.8	3.81	−0.41	10.8	160.6–164.3	2	hotspot14	2.0
*cochr2.3*	COchr2	8	*trans*	1.9	3.64	0.31	10.6	0–6.8	46	hotspot54	203.0
*cochr3.1*	COchr3	2	*trans*	53.4	2.88	0.46	9.5	50.5–56.2	2	near to hotspot18	5.2
*cochr3.2*	COchr3	2	*trans*	80.0	3.33	−0.33	11.0	74.7–81.0	5	hotspot20	5.6
*cochr3.3*	COchr3	2	*trans*	85.5	3.04	−0.30	10.0	81.9–87.6	290	near to hotspot20	721.9
*cochr4.1*	COchr4	4	*cis-prone*	45.0	8.32	−0.96	29.1	44.5–45.1	112	/	396.4
*cochr4.2*	COchr4	4	*cis-prone*	56.0	8.46	0.85	24.1	55.0–58.0	3	hotspot38	6.2
*cochr6.1*	COchr6	6	*cis-prone*	10.1	4.59	−0.72	15.1	9.9–14.4	4	hotspot46	23.5
*cochr7.1*	COchr7	7	*cis-prone*	2.8	5.82	−0.25	21.4	1.6–4.1	247	/	836.5
*cochr8.1*	COchr8	1	*trans*	97.3	3.35	−0.34	9.3	94.2–99.9	44	near to hotspot10	117.3
*cochr8.2*	COchr8	1	*trans*	105.5	3.40	−0.34	8.7	103.6–113.2	11	hotspot10	37.5
*hco.1*	HCO	1	/	5.8	3.26	−0.63	9.9	0–9.2	4	hotspot1	9.3
*hco.2*	HCO	2	/	75.6	3.73	−0.66	11.0	69.8–80.5	4	hotspot20	5.8
*tco.1*	TCO	2	/	81.9	3.10	−0.84	10.6	80.5–85.0	197	near to hotspot20	467.7

Note: ^a^ CI: confidence intervals of QTLs. ^b^ No. of genes: the number of genes in the confidence intervals of QTLs. ^c^ Association with hotspots: the association of the physical location of QTLs and crossover hotspots.

## Data Availability

Data of this study are included in the article or Supplementary Materials.

## Supplementary Materials

The following are available online at https://www.mdpi.com/article/10.3390/jof7121076/s1, Figure S1: Variation of crossovers among the 110 SSIs. Figure S2: Significant differences of TCO and HCO among the SSIs with different MAT-A loci. Table S1: Crossovers in the 110 single spore isolates. Table S2: Table S2. Crossovers and lengths of nine chromosomes in *L. edodes*. Table S3: Distribution of crossover hotspot regions in *L. edodes* genome. Table S4: Associations of corssovers and genes regulating fruiting body-related traits. Table S5: Genes in confidence interval of QTLs for crossover variation in *L.edodes*. Supplementary File S1: findCrossover.py.
