# Supplementary material for: Chromosome-Wide Characterization of Intragenic Crossover in Shiitake Mushroom, Lentinula edodes
Source: J Fungi (Basel). 2021 Dec 15;7(12):1076. doi: 10.3390/jof7121076 (PMC8704546; doi:10.3390/jof7121076)
Supplement: Supplementary file 1 [file jof-07-01076-s001.zip › Supplementary Figures.pdf]

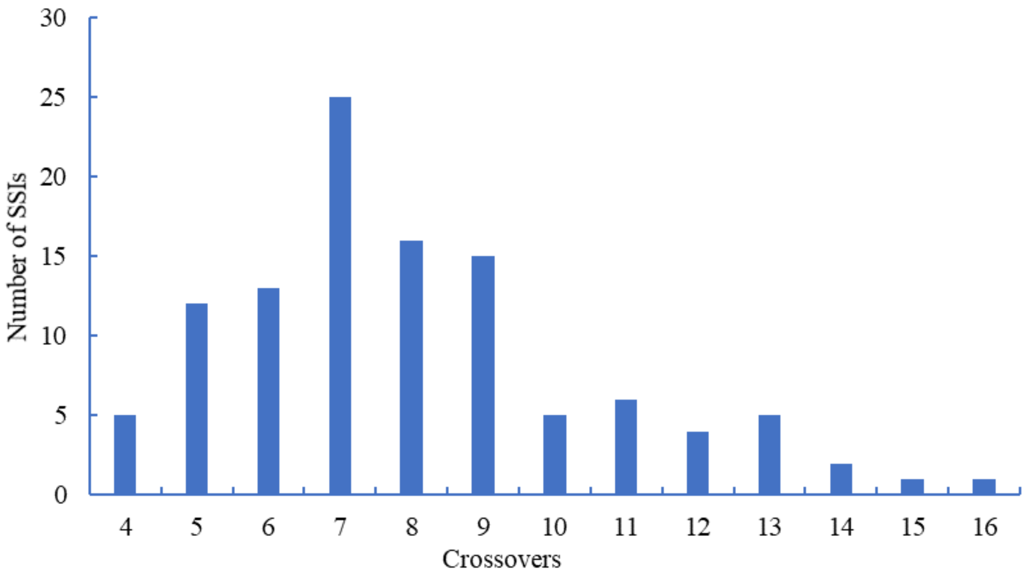

Figure S1. Variation of crossovers among the 110 SSIs.

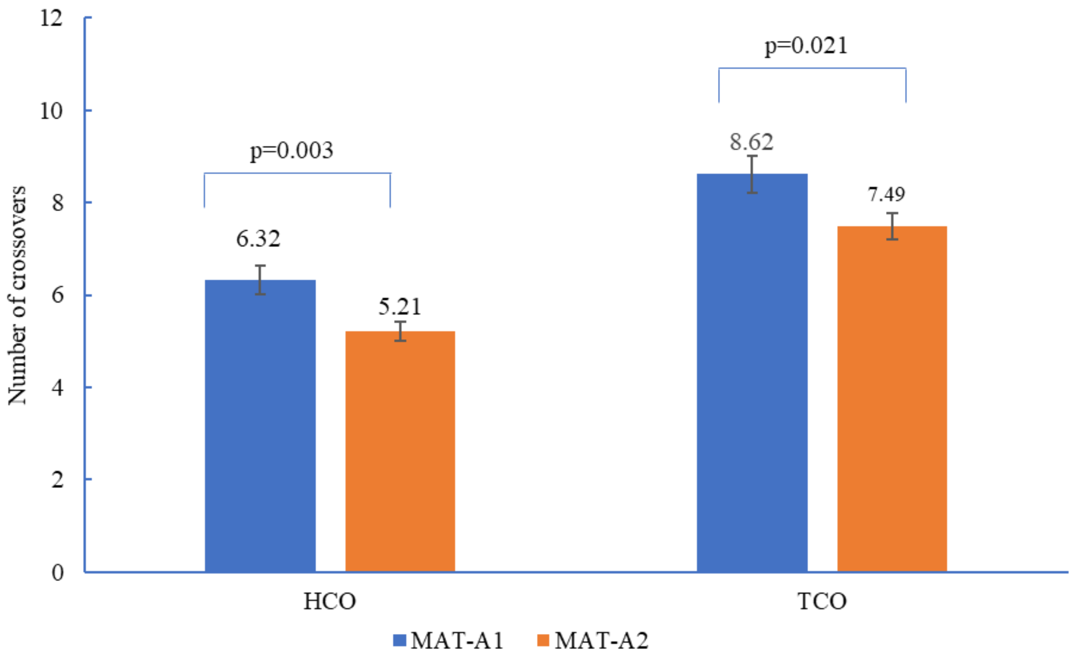

Figure S2. Significant differences of TCO and HCO among the SSIs with different MAT-A loci.
